# Prevalence and factors associated with lipid-lowering medications use for primary and secondary prevention of cardiovascular diseases among Malaysians: the REDISCOVER study

**DOI:** 10.1186/s12889-022-12595-1

**Published:** 2022-02-04

**Authors:** Noorhida Baharudin, Mohamed-Syarif Mohamed-Yassin, Aqil Mohammad Daher, Anis Safura Ramli, Nor-Ashikin Mohamed Noor Khan, Suraya Abdul-Razak

**Affiliations:** 1 Department of Primary Care Medicine, Faculty of Medicine, Universiti Teknologi MARA, Selayang Campus, Jalan Prima Selayang 7, 68100 Batu Caves, Selangor Malaysia; 2 Department of Community Medicine, School of Medicine, International Medical University, Bukit Jalil, 57000 Kuala Lumpur, Malaysia; 3 Institute of Pathology, Laboratory and Forensic Medicine (I-PPerForM), Universiti Teknologi MARA, Sungai Buloh Campus, Jalan Hospital, 47000 Sungai Buloh, Selangor Malaysia; 4 Department of Physiology, Faculty of Medicine, Universiti Teknologi MARA, Sungai Buloh Campus, Jalan Hospital, 47000 Sungai Buloh, Selangor Malaysia; 5 Hospital Universiti Teknologi MARA (HUiTM), 42300 Bandar Puncak Alam, Selangor Malaysia; 6 Cardio Vascular and Lungs Research Institute (CaVaLRI), Universiti Teknologi MARA, Sungai Buloh Campus, Jalan Hospital, 47000 Sungai Buloh, Selangor Malaysia

**Keywords:** Lipid-lowering medication, Statin, Primary prevention, Secondary prevention, Malaysia

## Abstract

**Background:**

Lipid-lowering medications (LLM) are commonly used for secondary prevention, as well as for primary prevention among patients with high global cardiovascular risk and with diabetes. This study aimed to determine the prevalence of LLM use among high-risk individuals [participants with diabetes, high Framingham general cardiovascular (FRS-CVD) score, existing cardiovascular disease (CVD)] and the factors associated with it.

**Methods:**

This is a cross-sectional analysis from the baseline recruitment (years 2007 to 2011) of an ongoing prospective study involving 11,288 participants from 40 rural and urban communities in Malaysia. Multiple logistic regression was used to identify characteristics associated with LLM use.

**Results:**

Majority (74.2%) of participants with CVD were not on LLM. Only 10.5% of participants with high FRS-CVD score, and 17.1% with diabetes were on LLM. Participants who were obese (OR = 1.80, 95% CI: 1.15–2.83), have diabetes (OR = 2.38, 95% CI: 1.78–3.19), have hypertension (OR = 2.87, 95% CI: 2.09–3.95), and attained tertiary education (OR = 2.25, 95% CI: 1.06–4.78) were more likely to be on LLM. Rural residents had lower odds of being on LLM (OR = 0.58, 95% CI: 0.41–0.82). In the primary prevention group, participants with high FRS-CVD score (OR = 3.81, 95% CI: 2.78–5.23) and high-income earners (OR = 1.54, 95% CI: 1.06–2.24) had higher odds of being on LLM.

**Conclusions:**

LLM use among high CVD-risk individuals in the primary prevention group, and also among individuals with existing CVD was low. While CVD risk factors and global cardiovascular risk score were positively associated with LLM use, sociodemographic disparities were observed among the less-educated, rural residents and low-income earners. Measures are needed to ensure optimal and equitable use of LLM.

## Background

Cardiovascular diseases (CVD) remain the leading cause of death, accounting for 32% of all deaths worldwide [1]. One of the risk factors for CVD is dyslipidaemia, which is highly prevalent among Malaysians, where 64% and 56.7% of Malaysian adults had elevated total cholesterol and low-density lipoprotein cholesterol (LDL-c), respectively [2]. Lipid-lowering medications (LLM), combined with therapeutic lifestyle changes are the cornerstone of dyslipidaemia management. Local and international guidelines recommend the use of LLM, specifically statins for secondary prevention, as well as for primary prevention among those with high risk to develop CVD [3–6]. All these guidelines consistently recommend stratifying patient’s CVD risk to guide LLM use, and to determine individual patient’s treatment target [3–6]. Among various risk-stratifying tools, the Framingham general cardiovascular (FRS-CVD) score is widely used, and has been validated among the multi-ethnic Malaysian population [7, 8]. Various literature advocates the use of LLM for patients with high CVD risk [3–6]. For Malaysians, high-risk individuals include those who have more than 20% of 10-year CVD risk calculated using the FRS-CVD score, those with diabetes or existing CVD [3, 4].

Despite recommendations from guidelines for prescription of LLM in these high-risk populations, its use in high-income and developed countries, as well as in developing countries varied [9–11]. Yusuf et al. reported that 66.5% of patients from the high-income countries, and only 3.3% from the low-income countries used statins, respectively [9]. Several clinical factors, such as diabetes and obesity had been identified as predictors for LLM use [12]. Inversely, several sociodemographic characteristics, such as ethnicity and the availability of health insurance had been found to be negatively associated with the use of LLM [13, 14]. In Malaysia, the prevalence of LLM use among individuals with hypercholesterolemia was previously reported [15], however its use among high CVD risk population has yet to be explored. Thus, the aim of this study was to determine the prevalence of LLM use for primary and secondary prevention of CVD among Malaysian adults, and the factors associated with it.

## Methods

### Study design and population

The REDISCOVER (*Re*spon*d*ing to *I*ncreasing *C*ardi*ov*ascular Dis*e*ase P*r*evalence) is an ongoing epidemiological prospective study with the duration of 15 years. The data was collected every 3 years, with the baseline collections from 2007 to 2011. This article presents the cross-sectional analysis of the primary data of RESDISCOVER study, which included sample of 11,288 respondents, aged 30 years and older from the baseline recruitments.

The REDISCOVER study involves 22 rural and 18 urban communities from five states across West and East Malaysia. The states representing West Malaysia were Selangor, Negri Sembilan, Pahang and Kelantan, while Sabah represented East Malaysia. These states were chosen to ensure adequate representation of the major ethnic groups in Malaysia. Major ethnic groups in West Malaysia, also known as Peninsular Malaysia are Malays, Indians and Chinese. Three states of West Malaysia (Selangor, Negri Sembilan and Pahang) have an adequate mixture of Malays, Chinese and Indians, while Kelantan population is predominantly Malay.

The major ethnic groups in Sabah (East Malaysia) were Kadazan-Dusun, Bajau, and Murut. These groups, along with other ethnic minorities in Sabah, are known as indigenous people. The sampling frame for this study were Malaysians aged 30 and above from the 40 communities.

### Subject recruitment

The participants were recruited in a standardized four-staged process; selecting the states, then communities, followed by the households within the communities, and then the individuals within the households. In each selected state and community, announcements and invitations were made through local community leaders. All household members aged 30 years and older received written invitations to attend screening sessions at local community centres. They were requested to fast for 8 hours in preparation for fasting blood sampling. The participants were screened for eligibility at the community centres, and written informed consent was obtained.

### Study procedure

All investigators and interviewers were trained on the study procedures. Researchers utilised standardised data collection forms that included demographic characteristics such as age, gender and educational attainment, as well as clinical details such as smoking status, history of diabetes mellitus, hypertension, CVD, and the use of LLM.

For anthropometric measurements, waist and hip circumferences were measured using a non-stretchable measuring tape while the participants stood in a relaxed position, with arms on the side. The measurements were recorded to the nearest 0.1 cm. Automatic digital blood pressure (BP) monitors (Omron HEM-757) were used to measure blood pressure after a five-minute rest. The BP on the right arm was measured twice, 2 minutes apart, with the participants in a seated position. The mean of the two measurements was taken as the BP reading for the participant.

Fasting venous blood samples were taken for lipid profiles [total cholesterol (TC), triglycerides (TG), high-density lipoprotein cholesterol (HDL-c), low-density lipoprotein cholesterol (LDL-c)] and plasma glucose level. The samples were centrifuged within 2 hours of collection, stored at the study sites in the minus 20-degree Celsius freezers, then transported frozen to the main laboratory at the Faculty of Medicine, Universiti Teknologi MARA, Malaysia. The samples were analysed using an automated clinical chemical analyzer (Cobas Integra 400 plus, Roche Diagnostic, Basel, Switzerland). The LDL-c was calculated using the Friedewald equation (for TG ≤ 4.5 mmol/L) [16].

### Variable definitions

Educational attainment was classified into four groups: no formal education, primary, secondary and tertiary. Primary education was defined as schooling between aged seven and 12 years old, while secondary education was schooling from 13 to 17 years old. Tertiary education was defined as the attainment of college or university qualification. Communities with a population of ≥10,000 were defined as urban, while those with populations of < 10,000 as rural [17]. Household income was classified according to the Department of Statistics Malaysia, where the income groups were classified into three categories: the bottom 40% (household earnings of Ringgit Malaysia (RM) ≤ 4849), medium 40% (household earnings of RM 4850–10,959) and top 20% (household earnings of RM ≥ 10,960) [18].

As for the clinical characteristics, CVD were defined as self-reported ischaemic heart disease and/or stroke. Smoking status was categorized as current smoker (had smoked any tobacco product in the last 5 years), non-smokers (had never smoked) and ex-smokers (had stopped smoking for more than 5 years). Body mass index (BMI) was classified as the following: underweight < 18.5 kg/m^2^, normal 18.5–22.9 kg/m^2^, overweight 23–27.4 kg/m^2^, or obese ≥27.5 kg/m^2^ [19, 20]. Abdominal obesity was defined as waist-hip ratio (WHR) of ≥0.90 for males and ≥ 0.85 for females [21].

The abnormal fasting lipid profile was defined according to the Malaysian Clinical Practice Guideline, which were: hypercholesterolaemia (TC > 5.2 mmol/L); hypertriglyceridaemia (TG > 1.7 mmol/L) and low HDL-c (HDL-c < 1 in males; < 1.2 mmol/L in females) [3]. This guideline also defines elevated LDL-c according to CVD risk profiles, where LLM is indicated when LDL-c > 1.8 mmol/L for very high-risk category, > 2.6 mmol/L for high-risk category and > 3.4 mmol/L for intermediate risk group [3]. For low-risk category, the guideline did not specify the LDL-c cut-off point for LLM, and recommended LLM therapy based on joint decision making between prescribers and patients; taking into account the risk and benefits of LLM therapy [3]. For the purpose of this study, the highest level, i.e., LDL-c > 3.4 mmol/L was chosen to define “elevated LDL-c”; as above this threshold, LLM is indicated for patients from intermediate, high risk, and very-high risk categories. Hypertension was defined as mean systolic blood pressure (BP) of ≥140 and/or mean diastolic BP ≥90 mmHg and/or self-reported hypertension and/or self-reported history of taking antihypertensive medications in the last month.

The participants were classified as having diabetes if they fulfilled any of the following criteria: fasting plasma glucose of ≥7.0 mmol/L and/or self-reported diabetes and/or taking any diabetes medications for the past 1 month. The Global CVD risk of participants without existing CVD was calculated using the 10-year FRS-CVD score [7]. Classifications were as follows: low risk (10-year FRS-CVD score <  10%), intermediate risk (10-year FRS-CVD score 10–20%) or high risk (10-year FRS-CVD score >  20%). Based on the Malaysian Clinical Practice Guidelines on Management of Dyslipidaemia, individuals with diabetes or a 10-year FRS-CVD score > 20% are classified as high risk, while those with existing CVD are automatically classified as very high-risk individuals [3]. For the purpose of this study, the three groups (diabetes, FRS-CVD score > 20% and existing CVD) will be collectively referred as high-risk individuals. Local guideline recommends LLM use at the outset among these high-risk individuals [3].

LLM use was defined as self-reported use of either one or any combinations of lipid lowering medications, such as statin, ezetimibe, or fibrate.

### Statistical analysis

The STATA software version 14 was used for statistical analysis [22]. The cases without specific variable data (missing data) were excluded from analysis for that variable only, using pairwise deletion. Descriptive statistics were presented as frequencies and percentages for categorical variables. For numerical variable, mean ± standard deviation (SD) was used for normally distributed data, or median with interquartile range (IQR) for non-normally distributed data, respectively. Chi-square test or Fisher’s exact test was used to test the difference in proportions. Simple and multiple logistic regressions were used to estimate the crude and adjusted odds ratio (OR) for the factors associated with the use of LLM. A *p*-value of less than 0.05 was considered statistically significant.

## Results

### Characteristics of the participants

Approximately 19,000 invitations were sent to household members above the age of 30 years to attend screening sessions at their local community centres. The response rates were between 60 and 70%. A total of 11,288 participants were included in the analysis. Their sociodemographic and clinical characteristics are presented in Table 1. The median age (IQR) was 52 (15) years. Majority of the participants were female (56.2%), and of Malay (72.5%) ethnicity. There was an almost equal distribution of urban (51.9%) and rural (48.1%) participants. Most of the participants (89.2%) were from the bottom 40% household income group. As for the clinical characteristics, 5.3% of participants had existing CVD. Among those without CVD, 23.4% were in the FRS-CVD high risk category.

**Table 1:** Sociodemographic and clinical characteristics, *N* = 11,288

Sociodemographic and clinical characteristics	
**Age (years), median (IQR)**	52 (15)
***Age groups (years) (n, %)***	
30–39	1233 (10.9)
40–49	3336 (29.6)
50–59	3606 (32.0)
≥ 60	3113 (27.6)
***Gender (n, %)***	
Male	4943 (43.8)
Female	6345 (56.2)
***Ethnicity (n, %)***	
Malay	8188 (72.5)
Chinese	1214 (10.8)
Indian	327 (2.9)
Indigenous	1559 (13.8)
***Education attainment (n^, %)***	
No formal education	1556 (15.3)
Primary school	2766 (27.0)
Secondary school	3929 (38.4)
Tertiary	1980 (19.3)
***Location (n, %)***	
Urban	5857 (51.9)
Rural	5431 (48.1)
***Occupation (n, %)***	
Homemaker/unemployed	4334 (38.4)
Semi-skilled	5181 (45.9)
Skilled workers	939 (8.3)
Managerial and Professional	834 (7.4)
***Household income group (n^, %)***	
Bottom 40% (≤ RM 4849)	4597 (89.2)
Middle 40% – Top 20% (≥ RM 4850)	554 (10.8)
***Smoking status (n^, %)***	
Non-smoker	8014 (75.6)
Previous smoker	1367 (12.9)
Current smoker	1224 (11.5)
***Body mass index (kg/m***^***2***^***) (n^, %)***	
Underweight (< 18.5)	392 (3.7)
Normal (18.5–22.9)	2493 (23.3)
Overweight (23–27.4)	4144 (38.7)
Obese (≥ 27.5)	3670 (34.3)
***Waist-hip ratio (n^, %)***	
Optimal/Normal	4834 (48.1)
High/Abdominal obesity (Male ≥0.90; female ≥0.85)	5221 (51.9)
***Comorbidities (n^, %)***	
Diabetes	1888 (17.7)
Hypertension	5409 (47.9)
Cardiovascular diseases (CVD)	596 (5.3)
***Dyslipidaemia subtypes (n^, %)***	
Hypercholesterolaemia	6897 (63.7)
High LDL-c	6065 (56.0)
Hypertriglyceridaemia	4084 (37.8)
Low HDL-c	3870 (35.7)
***Self-reported lipid-lowering medication use (n^, %)***	
No	9793 (92.4)
Yes	806 (7.6)
10-year Framingham general cardiovascular risk (FRS-CVD) categories (n*, %)	
Low risk < 10%	5939 (55.6)
Intermediate risk 10–20%	2252 (21.1)
High risk > 20%	2501 (23.4)

n^ is not equal to 11,288 due to missing values

n* (for participants without CVD) = 10,692

Ringgit Malaysia (RM) = Malaysian currency

### Prevalence of lipid-lowering medications use among high-risk participants

Table 2 presents the use of LLM among high-risk participants. In each group, the majority of participants were not on LLM. Out of those with existing CVD, 74.2% were not on LLM for secondary prevention of CVD. As for participants with diabetes, only 17.1% were on LLM. Among those with high FRS-CVD risk score, 89.5% of participants were not on LLM.

**Table 2:** Lipid-lowering medication (LLM) use among high-risk participants

	Cardiovascular diseases (CVD), ***n*** = 596			Diabetes, ***n*** = 1799			10-year Framingham general cardiovascular risk (FRS-CVD) > 20%, n^ = 2478		
	Not on LLM, n (%)	On LLM, n (%)	***p***-value	Not on LLM, n (%)	On LLM, n (%)	*p*-value	Not on LLM, n (%)	On LLM, n (%)	*p*-value
***Overall (n, %)***	439 (74.2)	153 (25.8)		1491 (82.9)	308 (17.1)		2217 (89.5)	261 (10.5)	
***Age groups (years)***									
30–39	15 (93.75)	1 (6.25)	0.026^*^	81 (93.10)	6 (6.90)	0.000^*^	92 (100.00)	0 (0)	0.001^*^
40–49	83 (83.00)	17 (17.00)		343 (88.17)	46 (11.83)		107 (84.25)	20 (15.75)	
50–59	136 (70.10)	58 (29.90)		549 (82.19)	119 (17.81)		685(87.93)	94 (12.07)	
≥60	205 (72.70)	77 (27.30)		518 (79.08)	137 (20.92)		1333 (90.07)	147 (9.93)	
***Gender***									
Male	238 (70.62)	99 (29.38)	0.024^*^	716 (80.72)	171 (19.28)	0.017^*^	1535 (89.50)	180 (10.50)	0.928
Female	201 (78.82)	54 (21.18)		775 (84.98)	137 (15.02)		682 (89.38)	81 (10.62)	
***Ethnicity***									
Malay	300 (72.99)	111 (27.01)	0.001^*^	1285 (83.93)	246 (16.07)	0.000^*^	1752 (90.17)	191 (9.83)	0.000^*^
Chinese	32 (65.31)	17 (34.69)		76 (66.67)	38 (33.33)		155 (74.16)	54 (25.84)	
Indian	10 (50.00)	10 (50.00)		71 (78.02)	20 (21.98)		47 (79.66)	12 (20.34)	
Indigenous	97 (86.61)	15 (13.39)		59 (93.65)	4 (6.35)		263 (98.50)	4 (1.50)	
***Education attainment***									
No formal education	106 (84.13)	20 (15.87)	0.000^*^	181 (89.60)	21 (10.40)	0.000^*^	450 (95.14)	23 (4.86)	0.000^*^
Primary school	153 (78.46)	42 (21.54)		462 (85.24)	80 (14.76)		779 (91.76)	70 (8.24)	
Secondary school	122 (72.62)	46 (27.38)		560 (83.09)	114 (16.91)		592 (86.68)	91 (13.32)	
Tertiary	42 (51.22)	40 (48.78)		218 (74.66)	74 (25.34)		279 (81.58)	63 (18.42)	
***Location***									
Urban	150 (59.52)	102 (40.48)	0.000^*^	762 (77.36)	223 (22.64)	0.000^*^	989 (84.10)	187 (15.90)	0.000^*^
Rural	289 (85.00)	51 (15.00)		729 (89.56)	85 (10.44)		1228 (94.32)	74 (5.68)	
***Occupation***									
Homemaker/unemployed	177 (70.52)	74 (29.48)	0.000^*^	643 (81.39)	147 (18.61)	0.004^*^	784 (87.99)	107 (12.01)	0.000^*^
Semi-skilled	220 (83.02)	45 (16.98)		615 (86.13)	99 (13.87)		1142 (92.69)	90 (7.31)	
Skilled workers	28 (63.64)	16 (36.36)		152 (82.16)	33 (17.84)		179 (82.87)	37 (17.13)	
Managerial and Professional	14 (43.75)	18 (56.25)		81 (73.64)	29 (26.36)		112 (80.58)	27 (19.42)	
***Household income group***									
Bottom 40% (≤ RM 4849)	226 (83.39)	45 (16.61)	**< 0.001**^**+**^	656 (87.58)	93 (12.42)	0.000^*^	1028 (92.45)	84 (7.55)	0.001^*^
Middle 40% – Top 20% (≥ RM 4850)	12 (50.00)	12 (50.00)		60 (73.17)	22 (26.83)		80 (82.47)	17 (17.53)	
***Smoking status***									
Non-smoker	283 (77.75)	81 (22.25)	0.001^*^	1068 (82.85)	221 (17.15)	0.000^*^	1295 (88.22)	173 (11.78)	0.003^*^
Previous smoker	64 (80.00)	16 (20.00)		209 (90.48)	22 (9.52)		324 (94.46)	19 (5.54)	
Current smoker	86 (62.77)	51 (37.23)		193 (75.98)	61 (24.02)		598 (89.66)	69 (10.34)	
***Body mass index (kg/m***^***2***^***)***									
Underweight (< 18.5)	24 (88.89)	3 (11.11)	0.000^*^	18 (90.00)	2 (10.00)	0.469	66 (98.51)	1 (1.49)	0.000^*^
Normal (18.5–22.9)	109 (90.08)	12 (9.92)		225 (85.55)	38 (14.45)		490 (93.51)	34 (6.49)	
Overweight (23–27.4)	155 (70.14)	66 (29.86)		524 (81.88)	116 (18.13)		871 (88.70)	111 (11.30)	
Obese (≥ 27.5)	145 (67.76)	69 (32.24)		708 (82.61)	149 (17.39)		765 (87.03)	114 (12.97)	
***Waist-hip ratio***									
Normal	149 (82.78)	31 (17.22)	0.001^*^	404 (87.45)	58 (12.55)	0.003^*^	637 (92.59)	51 (7.41)	0.001^*^
Abdominal obesity (Male ≥0.90; female ≥0.85)	263 (69.21)	117 (30.79)		996 (81.37)	228 (18.63)		1442 (88.14)	194 (11.86)	
***Dyslipidaemia subtypes***									
Hypercholesterolaemia	250 (76.45)	77 (23.55)	0.180	992 (85.81)	164 (14.19)	0.000^*^	1593 (90.36)	170 (9.64)	0.023^*^
High LDL-c	215 (77.62)	62 (22.38)	0.081	846 (87.31)	123 (12.69)	0.000^*^	1423 (91.39)	134 (8.61)	0.000^*^
Hypertriglyceridaemia	175 (72.92)	65 (27.08)	0.506	809 (84.36)	150 (15.64)	0.061	1143 (88.67)	146 (11.33)	0.180
Low HDL-c	168 (78.14)	47 (21.86)	0.106	635 (87.23)	93 (12.77)	0.000^*^	909 (91.36)	86 (8.64)	0.012^*^

Abbreviation: *LLM* Lipid-lowering medications

+ Fisher’s exact test, *p* < 0.001

* Chi-square test, *p* < 0.05

^ for participants without CVD

### Factors associated with lipid-lowering medications use

The factors associated with LLM use among all participants are shown in Table 3. Those who received secondary education (OR = 2.46, 95% CI: 1.29–4.70) and tertiary education (OR = 2.25, 95% CI: 1.06–4.78) were more likely to be on LLM, compared to those with no formal education. Participants residing in rural areas had less odds of being on LLM (OR = 0.58, 95% CI: 0.41–0.82) compared to urbanites.

**Table 3:** Factors associated with lipid-lowering medications use

	Crude OR (95% CI)	*p*-value	Adjusted OR (95% CI)	*p*-value
***Age groups (years)***				
30–39	1		1	
40–49	2.00 (1.31–3.06)	0.001*	0.97 (0.53–1.78)	0.92
50–59	4.47 (2.98–6.70)	0.000*	1.57 (0.86–2.87)	0.14
≥60	4.99 (3.32–7.49)	0.000*	1.69 (0.88–3.27)	0.12
***Gender***				
Male	1		1	
Female	0.75 (0.65–0.87)	0.000*	1.00 (0.72–1.40)	0.98
***Ethnicity***				
Malay	1		1	
Chinese	2.17 (1.80–2.61)	0.000*	1.49 (0.91–2.44)	0.11
Indian	2.67 (1.95–3.66)	0.000*	1.25 (0.66–2.35)	0.49
Indigenous	0.40 (0.30–0.55)	0.000*	1.34 (0.77–2.31)	0.30
***Education attainment***				
No formal education	1		1	
Primary school	1.52 (1.14–2.03)	0.004*	1.10 (0.58–2.11)	0.770
Secondary school	1.86 (1.42–2.44)	0.000*	2.46 (1.29–4.70)	0.006*
Tertiary	2.78 (2.10–3.68)	0.000*	2.25 (1.06–4.78)	0.035*
***Location***				
Urban	1		1	
Rural	0.34 (0.29–0.40)	0.000*	0.58 (0.41–0.82)	0.002*
***Occupation***				
Homemaker/unemployed	1		1	
Semi-skilled	0.66 (0.56–0.78)	0.000*	0.87 (0.58–1.31)	0.516
Skilled workers	1.16 (0.91–1.48)	0.239	1.12 (0.66–1.90)	0.680
Managerial and Professional	1.56 (1.23–1.97)	0.000*	1.42 (0.78–2.60)	0.250
***Household income group***				
Bottom 40% (≤ RM 4849)	1		1	
Middle 40% – Top 20% (≥ RM 4850)	2.80 (2.12–3.69)	0.000*	1.57 (0.99–2.50)	0.054
***Smoking status***				
Non-smoker	1		1	
Previous smoker	0.59 (0.45–0.77)	0.000*	0.56 (0.33–0.95)	0.032*
Current smoker	1.70 (1.40–2.06)	0.000*	1.18 (0.81–1.70)	0.386
***Body mass index (kg/m***^***2***^***)***				
Normal (18.5–22.9)	1		1	
Underweight (< 18.5)	2.65 (1.23–5.73)	0.013*	0.33 (0.04–2.44)	0.274
Overweight (23–27.4)	4.71 (2.21–10.03)	0.000*	1.71 (1.11–2.64)	0.015*
Obese (≥ 27.5)	5.65 (2.65–12.03)	0.000*	1.80 (1.15–2.83)	0.010*
***Waist-hip ratio***				
Normal	1		1	
Abdominal obesity (Male ≥0.90; female ≥0.85)	1.96 (1.67–2.29)	0.000*	1.40 (1.03–1.92)	0.032*
***Diabetes***				
No	1		1	
Yes	3.44 (2.96–4.01)	0.000*	2.38 (1.78–3.19)	0.000*
***Hypertension***				
No	1		1	
Yes	2.98 (2.54–3.50)	0.000*	2.87 (2.09–3.95)	0.000*
***Cardiovascular diseases (CVD)***				
No	1		1	
Yes	4.99 (4.09–6.10)	0.000*	3.30 (2.26–4.84)	0.000*
***Hypercholesterolaemia***				
No	1		1	
Yes	0.85 (0.73–0.98)	0.029*	1.27 (0.83–1.95)	0.274
***High LDL-c***				
No	1		1	
Yes	0.70 (0.60–0.81)	**0.000**	0.66 (0.44–0.98)	0.037*
***Hypertriglyceridaemia***				
No	1		1	
Yes	1.24 (1.07–1.44)	**0.005**	1.11 (0.82–1.50)	0.496
***Low HDL-c***				
No	1		1	
Yes	0.77 (0.65–0.90)	**0.001**	0.72 (0.53–0.99)	0.039*

Abbreviation: *OR* Odds ratio

Variables included in multiple logistic regression model: age, gender, ethnicity, education, location (urban/rural), occupation, household income, smoking status, body mass index, waist-hip ratio, diabetes, hypertension, CVD, dyslipidaemia subtypes (hypercholesterolaemia, high LDL-c, hypertriglyceridaemia, low HDL-c. No significant interactions

*Significant at *p* < 0.05

Participants who had CVD risk factors, such as obesity (OR = 1.80, 95% CI: 1.15–2.83), diabetes (OR = 2.38, 95% CI: 1.78–3.19), and hypertension (OR = 2.87, 95% CI: 2.09–3.95) were more likely to be on LLM. As for secondary prevention, those with existing CVD had increased odds of being on LLM (OR = 3.30, 95% CI: 2.26–4.84). Participants with elevated LDL-c (OR = 0.66, 95% CI: 0.44–0.98) and low levels of HDL-c (OR = 0.72, 95% CI: 0.53–0.99) were less likely to be on LLM (OR = 0.66, 95% CI: 0.44–0.98).

The FRS-CVD score was calculated for the primary prevention group, and included in a separate multiple logistic regression model. Table 4 presents factors associated with LLM use among the primary prevention group. After adjusting for socio-demographic confounders, participants in the high-risk category had higher odds of being on LLM (OR = 3.81, 95% CI: 2.78–5.23), compared to those in the low-risk category. Similar to the model that included the overall cohort (primary and secondary prevention groups), this model (primary prevention group only) found that education level and locality (urban versus rural) were significant determinants of LLM prescription. In addition, income group was also a significant factor in this model (primary prevention group), where those who earned equal or more than RM 4850 were 1.5 times more likely to be on LLM, compared to those from the lower income group (OR = 1.54, 95% CI: 1.06–2.24).

**Table 4:** Factors associated with lipid-lowering medications use for primary prevention of CVD^

	Crude OR (95% CI)	*p*-value	Adjusted OR (95% CI)	*p*-value
***Ethnicity***				
Malay	1		1	
Chinese	2.42 (1.98–2.95)	0.000*	1.38 (0.89–2.16)	0.154
Indian	2.69 (1.91–3.80)	0.000*	1.31 (0.70–2.43)	0.396
Indigenous	0.34 (0.23–0.50)	0.000*	1.01 (0.58–1.77)	0.960
***Education attainment***				
No formal education	1		1	
Primary school	1.63 (1.17–2.29)	0.004*	1.50 (0.73–3.08)	0.274
Secondary school	2.16 (1.57–2.97)	0.000*	**3.24 (1.61–6.52)**	0.001*
Tertiary	3.08 (2.21–4.27)	0.000*	**3.02 (1.37–6.66)**	0.006*
***Location***				
Urban	1		1	
Rural	0.32 (0.27–0.38)	0.000*	**0.56 (0.40–0.79)**	0.001*
***Occupation***				
Homemaker/unemployed	1		1	
Semi-skilled	0.69 (0.58–0.83)	0.000*	0.78 (0.53–1.15)	0.212
Skilled workers	1.18 (0.90–1.55)	0.224	0.93 (0.55–1.56)	0.784
Managerial and Professional	1.58 (1.22–2.04)	0.001*	1.03 (0.58–1.85)	0.920
***Household income group***				
Bottom 40% (≤ RM 4849)	1		1	
Middle 40% – Top 20% (≥ RM 4850)	2.81 (2.08–3.79)	0.000*	**1.54 (1.06–2.24)**	0.025*
***10-year Framingham general cardiovascular risk (FRS-CVD) categories***				
Low risk < 10%	1		**1**	
Intermediate risk 10–20%	1.76 (1.43–2.16)	0.000*	**2.50 (1.78–3.49)**	0.000*
High risk > 20%	2.61 (2.17–3.14)	0.000*	**3.81 (2.78–5.23)**	0.000*

Abbreviation: *OR* Odds ratio

**^** include participants without CVD, *n* = 10,692

Variables included in multiple logistic regression model: ethnicity, education, location (urban/rural), occupation, household income, 10-year Framingham general cardiovascular risk (FRS-CVD) categories. No significant interactions

*Significant at *p* < 0.05

## Discussion

This study discovered that the majority of participants with high CVD risk were not on LLM. Although guidelines support its use for secondary and primary prevention of CVD among these patients [3–6], a high proportion of participants from this study were not on any LLM. This study discovered that only 25.8% of participants with existing CVD were on LLM for secondary prevention. Yusuf et al. also discovered similar findings, where only 14.6% of their study population were on LLM for secondary prevention [9]. A worrying trend was also observed in European countries, where 12% of patients were not on LLM at hospital discharge following admission for cardiac procedure or event, and the proportion had increased to 16% during follow up 1 year later [23]. Another Malaysian study showed a much higher utilisation of statin for secondary prevention (99.1%); however, it was a small study conducted in a primary care centre; which findings are not generalisable to the general population [24].

In terms of primary prevention, diabetes has been identified as a major cardiovascular risk, and was found in 17.7% of this study population. The prevalence of diabetes in this study was comparable to the Malaysian Health and Morbidity Survey, which has been increasing in trend (13.4% in 2015 and 18.3% in 2019) [25]. Various guidelines recommend intensive lipid-lowering therapy with strict LDL-c target among these group of patients [3–6]. In this study, only 17.1% of participants with diabetes were using LLM. Another smaller study in Malaysia demonstrated a better result, where 87.6% of their patients with diabetes received LLM [26]. This study however; was conducted at four primary care clinics in one state in Malaysia, thus the results need to be interpreted with caution due to the potential lack of generalizability to the general population [26]. The finding of low LLM utilisation was also observed in Ghana, another developing country, where only 16.8% of patients with diabetes were on LLM [27]. The outcomes from developed countries demonstrated greater compliance with clinical guidelines where 57.8% of patients with diabetes were prescribed statin in Ireland [28]. Similar finding was also observed in Canada, where 72.1% of type 1 diabetes patients were on statins [29]. CVD remains the leading cause of morbidity and mortality among patients with diabetes, where they have a two-fold excess risk of developing a wide range of vascular disorders [30]. A metanalysis by the Cholesterol Treatment Trialist (CTT) collaborators reported a 23% reduction in 5-year CVD events for every unit reduction in LDL-c level among patients with diabetes on statin therapy [31]. While these findings were not without controversy [32], clinical practice guidelines have consistently recommended LLM for patients with high CVD risk, including those with diabetes [3–6]. Thus, LLM prescription is appropriate in this group of patients to comply with these recommendations.

In addition to the above indications, patients with a combination of multiple CVD risk factors should also be on LLM [3–6]. Local Malaysian guidelines recommend stratifying each patient’s CVD risk using the Framingham general cardiovascular risk (FRS-CVD) algorithm [3, 4]. The algorithm enables physicians to assess each patient’s 10-year CVD risks, specifically for coronary artery disease, stroke, peripheral vascular disease and heart failure [7]. Patients who score more than 20% are classified as high-risk of developing CVD in the next 10  years [7]. These patients require LLM from the outset, in conjunction with strict therapeutic lifestyle interventions [3]. This study however, discovered that the majority of participants who had high FRS-CVD risk scores were not on LLM (89.5%). Similar finding was also seen in Ireland, where less than a quarter of this group of patients were on LLM [28]. The results from the United States were better; albeit still suboptimal. Approximately half (56.8%) of the patients who had high CVD risk were not on any LLM [10].

This study discovered that various clinical characteristics and comorbidities were associated with LLM use. Participants with obesity, diabetes or hypertension were more likely to be on LLM. Similar findings were also observed in other studies where these comorbidities were associated with the use of LLM [11, 12, 33, 34]. As these comorbidities are risk factors for CVD, it is appropriate for these patients to be on LLM, compared to those without these comorbidities. As for other CVD risk factors, the proportion of LLM use was highest among smokers, and lowest among previous smokers in the secondary prevention group and in those with diabetes. Previous smokers were also less likely to be on LLM, compared to non-smokers (OR = 0.56, 95% CI: 0.33–0.95). Our findings may be explained by previous literature which discovered that previous smokers were less adherent to medications compared to non-smokers [35].

This study stratified the CVD risk of the participants in the primary prevention group according to the FRS-CVD risk algorithm, and discovered that those in the high-risk category had almost four times the odds of being on LLM, compared to those from the low-risk group. This finding was reassuring, consistent with the recommendation from the guidelines [3–6].

In terms of secondary prevention, this study discovered that participants with existing CVD had increased odds of being on LLM, which concurred with findings by Bertolotti and Neutel et al. [12, 34]. While this is encouraging, the use of LLM for secondary prevention in this study (25.8%) was still low. Therefore, rigorous efforts should be carried out to optimize LLM use in this population.

This study also discovered sociodemographic disparities in LLM use. Participants were more likely to be on LLM if they resided in urban areas. This finding was also shared by Wu et al., which found that the populations who resided in a more deprived area had lower odds of being on LLM [11]. A study conducted in rural Malaysia reported that 24.8% of their study population experienced unmet medical needs [36]. Rural residents also suffer from poorer health status, and limitations in their activities due to illness and have an increased likelihood of being diagnosed with a serious disease [37]. Furthermore, rural areas had a higher rate of CVD mortality [38]. While various factors may contribute to this, under-utilization of CVD medications such as LLM should be avoided in order to reduce CVD morbidity and mortality in this population.

In this study, education level was a significant sociodemographic determinant of LLM use. Those with secondary and tertiary education were approximately twice more likely to be on LLM. Neutel et al. also found that people with high school education were more likely to be on LLM [12]. This finding may be explained by previous literature which found that higher levels of education were associated with a variety of positive health outcomes, including lower mortality, morbidity, and improved health behaviours and awareness [39]. Furthermore, positive health-seeking behaviours were found to be predictors for LLM use in some studies. According to these studies, those who frequently visit their physician were more likely to be on LLM [12, 28].

As for other sociodemographic determinants, this study discovered that participants with higher income were more likely to be on LLM in the primary prevention group. This finding was also observed in China where the low-income earners demonstrated lower rates of treatment and control of dyslipidaemia [40]. The Malaysian healthcare system offers free public healthcare which include LLM. While LLM is not indicated for all patients in the primary prevention group, this finding highlights the need for prescribers to examine their practices. This is to ensure that LLM is prescribed appropriately based on patient’s clinical indications so that those who are medically eligible, but socio-demographically disadvantaged, have equal access to LLM, especially when common LLM, such as statins are free to all Malaysians who attend public healthcare facilities.

### Strength and limitation

The main strength of this study is the large sample size with good response rate (60–70%) from various parts of East and West Malaysia, which provided good representation of the population. Although the response rate is acceptable, recruitment via voluntary participation may introduce non-response bias. This is likely minimal as the characteristics of our study population were comparable to another Malaysian study with similar setting, where 52.4% of their study population were females, and 42.3% were rural residents [15], whereas our study comprised of 56.2% females and 48.1% rural residents. While another large local population study used non-fasting, finger-prick capillary blood lipids [15], this study demonstrated robustness of laboratory measurements by using fasting venous blood sampling as recommended by the Malaysian guideline [3], which is commendable for a large epidemiological study.

This study had several limitations. The participants were recruited from health screening sessions at the local community centres. Self-reporting was used to collect several variables, like LLM use, which may have led to recall bias. Although participants were reminded to bring their medications to these sessions for verification by the interviewers, the vast majority did not. Interviewers were trained to ask specific questions to clarify the information provided by the participants, such as the reason for their LLM use, to minimise recall bias. Data on contraindications to LLM were not available in this study. Therefore, those high-risk individuals who were not on LLM may be contraindicated to receive LLM.

The participants who attended these voluntary community health screening events may have a greater awareness of their health which may also help to minimise recall bias. This may also explain the findings of our study which had lower prevalence of smokers (11.5%) compared to the results from the Malaysian National Health and Morbidity Survey (21.3%) [25]. Another limitation includes overrepresentation of Malays in this study. Malays, Chinese and Indians represent 63.1, 24.6 and 7.3% of Malaysia’s population, respectively [17], while this study comprised 72.5% of Malay participants. The use of survey weight could be employed to overcome this limitation, nevertheless it was not done in this study. Finally, the cross-sectional analysis of this study would only reflect the association between participant characteristics and LLM use, not the causal relationship. Thus, the results should be interpreted in this context.

### Implication for future research and clinical practice

The REDISCOVER study provided an insight to healthcare providers and policy makers regarding the widespread underutilisation of LLM among Malaysian adults, especially those who are in the high-risk categories. Urgent public health measures are required to address this issue. Two main possible reasons for the underutilisation could be due to prescriber factors or individual patient factors. A Malaysian study showed that while the majority of clinicians used dyslipidaemia guidelines, there were wide variation in their practice with some clinicians did not prescribe based on the guidelines [41]. Furthermore, another study reported a high proportion of therapeutic inertia for LLM (61%), which resulted in lower odds of achieving LDL-c target [42]. Various factors have been proposed to contribute to treatment inertia, including physician’s fear of side effects, as well as the availability of appropriate medications [42]. Inappropriate prescriptions were also observed in a previous study in the United Kingdom where most patients were initiated on statins without global CVD risk assessments, and some overtreatment also occurred in the low-risk patients [43]. A Malaysian study reported better results where 79.7% of clinicians performed risk-scoring prior to statins prescription [41]. In order to resolve the possible knowledge gap that could have led to inappropriate LLM use, the results of this study should be conveyed to prescribers via continuous medical education programmes. With regards to the individual patient factors, the findings of this study on sociodemographic disparities provide policymakers with an insight to ensure equitable access to LLM for individuals with lesser formal education, low-income earners and rural residents. In terms of future research, certain variables that could influence the use of LLM, such as medication adherence, health belief towards LLM and health literacy should also be included, to help identify patient factors contributing to suboptimal LLM use. Future research should also include collaboration with the participants’ health care providers to verify the type of LLM prescribed which will provide more credence to the study. Factors that influence prescribing of LLM among health care providers, such as contraindication to LLM, their knowledge on guideline recommendations and personal belief towards LLM should also be explored.

## Conclusions

This study highlights the low utilisation of LLM among high CVD-risk individuals in the primary prevention group, and also among individuals with existing CVD. While several CVD risk factors including high global cardiovascular risk stratification were found to be associated with increased likelihood of LLM use, sociodemographic disparities were also observed among the less-educated, low-income earners and rural residents. Intervention is required to address these findings.

## Data Availability

The datasets used during the current study are stored at the Centre for Translational Research and Epidemiology (CenTRE), Faculty of Medicine, Universiti Teknologi MARA, Malaysia. Datasets may be shared upon reasonable request to the corresponding author and subjected to the data protection regulation.

## Abbreviations

LLM: Lipid-lowering medications
CVD: Cardiovascular diseases
FRS-CVD: Framingham general cardiovascular
TC: Total cholesterol
TG: Triglycerides
LDL-c: Low-density lipoprotein cholesterol
HDL-c: High-density lipoprotein cholesterol
RM: Ringgit Malaysia
BMI: Body mass index
WHR: Waist-hip ratio
BP: Blood pressure
SD: Standard deviation
IQR: Interquartile range
OR: Odd ratio
CI: Confidence interval

## References

[1] World Health Organisation. Cardiovascular Diseases (CVDs). https://www.who.int/news-room/fact-sheets/detail/cardiovascular-diseases-(cvds). Accessed 16 June 2021.

[2] Mohamed-Yassin MS, Baharudin N, Daher AM, Abu-Bakar N, Ramli AS, Abdul-Razak S, et al. High prevalence of dyslipidaemia subtypes and their associated personal and clinical attributes in Malaysian adults: the REDISCOVER study. BMC Cardiovasc Disord. 21(1):149, Mar 23 2021. 10.1186/s12872-021-01956-0. 10.1186/s12872-021-01956-0 PMC7988996 33757445

[3] Clinical practice guidelines Management of Dyslipidaemia: Ministry of Health Malaysia. 2017. http://www.acadmed.org.my/. Accessed 16 June 2021.

[4] Clinical Practice Guidelines on Primary & Secondary Prevention of Cardiovascular Disease 2017: Ministry of Health Malaysia. 2017. http://www.acadmed.org.my/. Accessed 16 June 2021.

[5] Mach F, Baigent C, Catapano AL, Koskinas KC, Casula M, Badimon L, et al. 2019 ESC/EAS guidelines for the management of dyslipidaemias: lipid modification to reduce cardiovascular risk. Eur Heart J, vol. 41, no. 1, pp. 111–188, Jan 1 2020, doi: 10.1093/eurheartj/ehz455. 10.1093/eurheartj/ehz455 31504418

[6] Grundy SM, Stone NJ, Bailey AL, Beam C, Birtcher KK, Blumenthal RS (2018). AHA/ACC/AACVPR/AAPA/ABC/ACPM/ADA/AGS/APhA/ASPC/NLA/PCNA guideline on the Management of Blood Cholesterol: a report of the American College of Cardiology/American Heart Association task force on clinical practice guidelines. J Am Coll Cardiol.

[7] D'Agostino RB, Vasan RS, Pencina MJ, Wolf PA, Cobain M, Massaro JM (2008). General cardiovascular risk profile for use in primary care: the Framingham Heart Study. Circulation.

[8] Chia YC, Gray SY, Ching SM, Lim HM, Chinna K (2015). Validation of the Framingham general cardiovascular risk score in a multiethnic Asian population: a retrospective cohort study. BMJ Open.

[9] Yusuf S, Islam S, Chow CK, Rangarajan S, Dagenais G, Diaz R (2011). Use of secondary prevention drugs for cardiovascular disease in the community in high-income, middle-income, and low-income countries (the PURE Study): a prospective epidemiological survey. Lancet.

[10] Sidebottom AC, Vacquier MC, Jensen JC, Bradley SM, Knickelbine T, Strauss C (2020). Trends in prevalence of guideline-based use of lipid-lowering therapy in a large health system. Clin Cardiol.

[11] Wu J, Zhu S, Yao GL, Mohammed MA, Marshall T (2013). Patient factors influencing the prescribing of lipid lowering drugs for primary prevention of cardiovascular disease in UK general practice: a national retrospective cohort study. PLoS One.

[12] Neutel CI, Morrison H, Campbell NR, de Groh M (2007). Statin use in Canadians: trends, determinants and persistence. Can J Public Health.

[13] Eastwood SV, Mathur R, Sattar N, Smeeth L, Bhaskaran K, Chaturvedi N (2021). Ethnic differences in guideline-indicated statin initiation for people with type 2 diabetes in UK primary care, 2006-2019: a cohort study. PLoS Med.

[14] Adedinsewo D, Taka N, Agasthi P, Sachdeva R, Rust G, Onwuanyi A (2016). Prevalence and factors associated with statin use among a nationally representative sample of US adults: National Health and nutrition examination survey, 2011-2012. Clin Cardiol.

[15] Mat Rifin H, Lourdes TG, Ab Majid NL, Abd Hamid HA, Hasani WS, Miaw YL, et al. Hypercholesterolemia Prevalence, Awareness, Treatment and Control among Adults in Malaysia: The 2015 National Health and morbidity survey, Malaysia. Glob J Health Sci. 2018;10(7).

[16] Friedewald WT, Levy RI, Fredrickson DS (1972). Estimation of the concentration of low-density lipoprotein cholesterol in plasma, without use of the preparative ultracentrifuge. Clin Chem.

[17] Department of Statistics Malaysia. "Population Distribution and Basic Demographic Characteristic Report 2010." Department of Statistics Malaysia. https://www.mycensus.gov.my/index.php/census-product/publication/census-2010/659-population-distribution-and-basic-demographic-characteristics-2010 Accessed 4 May 2021.

[18] Department of Statistics Malaysia. Household Income & Basic Amenities Survey Report 2019. Department of Statistics Malaysia. https://www.dosm.gov.my/v1/index.php?r=column/cthemeByCat&cat=120&bul_id=TU00TmRhQ1N5TUxHVWN0T2VjbXJYZz09&menu_id=amVoWU54UTl0a21NWmdhMjFMMWcyZz09. Accessed 16 June 2021.

[19] Clinical practice guidelines on Management of Obesity, Putrajaya: Ministry of Health Malaysia. 2004. http://www.acadmed.org.my/. Accessed 16 June 2021.

[20] World Health Organization. The Asia-Pacific perspective : redefining obesity and its treatment. https://apps.who.int/iris/handle/10665/206936. Accessed 23 Nov 2021.

[21] World Health Organization (2008). Waist circumference and waist-hip ratio report of a WHO expert consultation.

[22] StataCorp. (2015). Stata statistical software: release 14.

[23] De Backer G, Jankowski P, Kotseva K, Mirrakhimov E, Reiner Z, Ryden L (2019). Management of dyslipidaemia in patients with coronary heart disease: results from the ESC-EORP EUROASPIRE V survey in 27 countries. Atherosclerosis.

[24] Baharudin N, Ahmad-Roslan AM, Mohamed-Yassin MS, Ramli AS, Zainal Abidin AN, Sahar NH. Gender disparity in the prescription of secondary prevention medications in a Malaysian primary care clinic. Malays Fam Physician. 16(2):2021. 10.51866/oa1080. 10.51866/oa1080 PMC8346747 34386162

[25] Institute for Public Health (IPH), National Institutes of Health, Ministry of Health Malaysia. 2020. National Health and morbidity survey (NHMS) 2019: Vol. I: NCDs – Non-Communicable Diseases: Risk Factors and other Health Problems http://iku.gov.my/images/IKU/Document/REPORT/NHMS2019/Report_NHMS2019-NCD_v2.pdf.

[26] Elnaem MH, Mohamed MH, Huri HZ, Shah AS (2019). Effectiveness and prescription pattern of lipid-lowering therapy and its associated factors among patients with type 2 diabetes mellitus in Malaysian primary care settings. Ther Clin Risk Manag.

[27] Sarfo FS, Ovbiagele B (2020). Prevalence and predictors of statin utilization among patient populations at high vascular risk in Ghana. J Neurol Sci.

[28] Byrne P, Cullinan J, Murphy C, Smith SM (2018). Cross-sectional analysis of the prevalence and predictors of statin utilisation in Ireland with a focus on primary prevention of cardiovascular disease. BMJ Open.

[29] Bai JW, Boulet G, Halpern EM, Lovblom LE, Eldelekli D, Keenan HA (2016). Cardiovascular disease guideline adherence and self-reported statin use in longstanding type 1 diabetes: results from the Canadian study of longevity in diabetes cohort. Cardiovasc Diabetol.

[30] Sarwar N, Gao P, Seshasai SR, Gobin R, Kaptoge S, Emerging Risk Factors Collaboration (2010). Diabetes mellitus, fasting blood glucose concentration, and risk of vascular disease: a collaborative meta-analysis of 102 prospective studies. Lancet.

[31] Baigent C, Blackwell L, Emberson J, Holland LE, Reith C, Bhala N, Cholesterol Treatment Trialists Collaboration (2010). Efficacy and safety of more intensive lowering of LDL cholesterol: a meta-analysis of data from 170,000 participants in 26 randomised trials. Lancet.

[32] Krumholz HM. Statins evidence: when answers also raise questions. BMJ 354, i4963, Sep 14 2016, doi: 10.1136/bmj.i4963. 10.1136/bmj.i4963 27628589

[33] Amor AJ, Ortega E, Gimenez M, Cofan M, Blanco J, Pane A, et al. Prevalence and factors associated with statin use in high-risk patients with type 1 diabetes from a specialized diabetes unit. Endocrinol Diabetes Nutr, vol. 66, no. 8, pp. 512–519, Oct 2019, doi: 10.1016/j.endinu.2019.02.004. 10.1016/j.endinu.2019.02.004 31000451

[34] Bertolotti M, Franchi C, Rocchi MB, Miceli A, Libbra MV, Nobili A (2017). Prevalence and determinants of the use of lipid-lowering agents in a population of older hospitalized patients: the findings from the REPOSI (REgistro POliterapie Societa Italiana di Medicina Interna) study. Drugs Aging.

[35] Mirahmadizadeh A, Khorshidsavar H, Seif M, Sharifi MH (2020). Adherence to medication, diet and physical activity and the associated factors amongst patients with type 2 diabetes. Diabetes Ther.

[36] Lim KK, Sivasampu S, Mahmud F (2017). Equity in access to health care in a rural population in Malaysia: a cross-sectional study. Aust J Rural Health.

[37] Fang H, Chen J, Rizzo JA (2009). Explaining urban-rural health disparities in China. Med Care.

[38] Sexton PT, Sexton TL (2000). Excess coronary mortality among Australian men and women living outside the capital city statistical divisions. Med J Aust.

[39] Higgins C, Lavin T, Metcalfe O. Health impacts of education: a review. Institute of Public Health in Ireland, 2008. https://www.drugsandalcohol.ie/11734/1/IPH_HealthImpactsofEducation.pdf. Accessed 16 June 2021.

[40] Pan L, Yang Z, Wu Y, Yin RX, Liao Y, Wang J (2016). The prevalence, awareness, treatment and control of dyslipidemia among adults in China. Atherosclerosis.

[41] Said AH, Chia YC (2017). Awareness, knowledge and practice of dyslipidaemia management among postgraduate primary care trainees in Malaysia: a cross-sectional study. BMJ Open.

[42] Chew BH, Hussain H, Supian ZA (2021). Is therapeutic inertia present in hyperglycaemia, hypertension and hypercholesterolaemia management among adults with type 2 diabetes in three health clinics in Malaysia? a retrospective cohort study. BMC Fam Pract.

[43] Finnikin S, Ryan R, Marshall T (2017). Statin initiations and QRISK2 scoring in UK general practice: a THIN database study. Br J Gen Pract.

[44] McKee M (2021). Khalid Yusoff: champion of excellence in Malaysian medical research. BMJ.

